# Effect of a 12-Week Strength Training Program on Muscle Strength Measures of Institutionalized Older Adults—A Pilot Study

**DOI:** 10.3390/healthcare12141428

**Published:** 2024-07-17

**Authors:** Bernardo Pereira, Diogo Monteiro, Rui Matos, Miguel Jacinto, Nuno Amaro, Raúl Antunes, Filipe Rodrigues

**Affiliations:** 1 ESECS—Polytechnic of Leiria, 2411-901 Leiria, Portugal; bernardo.pereira.ft@gmail.com (B.P.); diogo.monteiro@ipleiria.pt (D.M.); rui.matos@ipleiria.pt (R.M.); miguel.s.jacinto@ipleiria.pt (M.J.); nuno.amaro@ipleiria.pt (N.A.); raul.antunes@ipleiria.pt (R.A.); 2 Research Center in Sport, Health, and Human Development (CIDESD), 5000-558 Vila Real, Portugal

**Keywords:** strength training, institutionalized, older adults, physical fitness

## Abstract

Institutionalized older adults are characterized by high levels of dependence and low levels of physical activity compared to those living in the community. This combination of factors leads to an increased risk of loss of muscle mass. Sarcopenia can be countered through strength training. The aim of the present study was to evaluate the effect of a strength training program on the physical fitness of institutionalized older adults. This study included a sample of 31 institutionalized older adults (20 females, 11 males) aged between 65 and 96 years (M = 81.64, SD = 8.67). Participants reported an average institutionalization duration of 2.43 years (SD = 2.20), ranging from half a year to ten years. A 12-week strength training program was implemented, with sessions held twice a week. Strength fitness was assessed through the following parameters: handgrip strength, upper limb muscular endurance, lower limb muscular endurance, agility and balance, body mass index, and waist circumference. The results demonstrated that a 12-week strength training program improved physical fitness in terms of lower limb muscular endurance, upper limb muscular endurance, agility, and dynamic balance (*p* < 0.05).

## 1. Introduction

According to the data from the 2021 Census conducted by the National Institute of Statistics [1], there were 2,423,639 individuals over 65 years old residing in Portugal, representing approximately 23.4% of the total population. Of this elderly population, around 146,000 individuals were institutionalized, accounting for approximately 6% of the elderly population. This figure rises to 10% for those over 80 years old [1,2].

Elderly individuals residing in care homes are generally characterized by high levels of dependency, leading to declines in various domains, including strength, balance, reaction time, coordination, and muscular and cardiovascular endurance [3]. Institutionalization exacerbates the trajectory of functional decline, which is more pronounced in the institutionalized population compared to those living in the community [4]. A meta-analysis by Kojima [5] showed that about 52% of institutionalized elderly individuals exhibit frailty syndrome, whereas only 10% of community-dwelling elderly exhibit this syndrome [5].

Frailty is a biological syndrome resulting from cumulative declines across multiple physiological systems, causing vulnerability to adverse events. Markers of frailty include muscle mass, strength, gait speed, balance, reaction time, coordination, and muscular and cardiovascular endurance. Multiple components must be present simultaneously for an individual to be clinically considered frail. Frailty in the elderly predicts the risk of hospitalization, falls, physical disability, and death [6]. The etiology of frailty is associated with chronic diseases, especially when two or more are present simultaneously. However, another pathway to frailty is through physiological changes due to aging, notably sarcopenia [6].

Sarcopenia is a syndrome characterized by the progressive loss of muscle mass, strength, and functionality [7]. Sarcopenia can be categorized based on its etiology. Primary sarcopenia is caused solely by the aging process. Secondary sarcopenia is subdivided into three categories, one of which is related to activity level, originating from a sedentary lifestyle, bed rest, deconditioning, or prolonged exposure to zero gravity [7]. Muscle mass begins to decline from age 50 at an annual rate of 1% to 5%. Strength training is a way to prevent, delay, or reverse sarcopenia, maximizing muscle strength in youth, maintaining it in adulthood, and attenuating its loss in old age [8].

Preventing sarcopenia is essential as it has high personal, social, and economic costs. In terms of human health, sarcopenia increases the risk of falls and fractures, affects daily living activities, is associated with cardiovascular and respiratory diseases, impacts cognition, leads to mobility losses, reduces quality of life, independence, and can result in death [7]. Financially, sarcopenia is costly for the healthcare system, increasing hospitalization risks and costs associated with prolonged hospital stays [8].

### 1.1. The Role of Strength Training

Strength training has consistently proven to be an effective and viable method to counteract sarcopenia and physical frailty. It reduces intramuscular fat infiltration, improves physical performance, and enhances muscle quality. Strong evidence indicates that strength training can mitigate the effects of aging on neuromuscular function and functional capacity, improving variables such as strength, muscle power, and muscle mass. Additionally, muscle development is associated with improved bone density, reduced disability, increased range of motion and flexibility, and a lower incidence of falls. Despite these benefits, strength training is not commonly practiced among the elderly [9,10].

For institutionalized elderly, the focus of strength training should be on improving or maintaining health, functional capacity, self-care, and independence. However, exercise has often been provided to this population as a recreational activity rather than a strategy for improving functional capacity, performance, and overall health [3]. Adequate levels of strength are necessary for the elderly to perform various daily tasks and reduce the likelihood of falls. Thus, muscle mass and strength are the main components of physical fitness. Strength training is the form of exercise that increases or maintains muscle mass and strength, helping the elderly preserve their independence and quality of life. It helps overcome muscle mass and strength loss, creates resilience, facilitates chronic disease management, and reduces physical vulnerability, preventing sarcopenia [11].

Most interventions studied apply multicomponent exercise programs, not only consisting of strength training but also incorporating balance, flexibility, and aerobic training [12,13]. Although these interventions improve physical fitness, it is challenging to determine which type of training specifically contributes to increasing muscle strength and reducing frailty. A study by Rydwik et al. [14] suggested that exercise in institutionalized elderly should focus on increasing strength and mobility. The study also indicated that this group might struggle with aerobic training due to insufficient muscle mass to support it. Balance training alone did not show significant results in improving balance post-intervention, making it pertinent to apply interventions focused exclusively on strength training. The lack of common exercise practice in care homes, despite the described benefits, suggests barriers to implementing exercise programs, such as low literacy, low motivation due to the perception of exercise as a waste of time, exercise-related myths, and social and environmental contexts [15].

It is crucial that exercise programs consider these factors and have low associated costs, be simple, and are easy to apply in groups to ensure that these factors do not become additional barriers to exercise [16]. Therefore, this study aims to evaluate the effect of a strength training program on physical fitness in institutionalized elderly individuals.

### 1.2. Current Research

A systematic review by Cadore [17] found that most studies demonstrating improvements in gait, balance, and fall risk used multicomponent training as an intervention. These programs are essential for maintaining mobility, musculoskeletal function, and the optimal functioning of other body systems: neurological, cardiovascular, respiratory, and endocrine. However, exercise prescription should consider that the ideal exercise intervention is the most time-efficient [18]. Studies that included strength training, either isolated or as part of multicomponent exercise programs, showed greater strength gains in elderly individuals with physical frailty or severe functional declines. The absence of changes in functional and strength outcomes in some multicomponent studies indicates that exercise prescription should be adapted to provide sufficient stimulus to improve functional capacity in frail individuals [17]. Therefore, it is pertinent to define an intervention exclusively focused on strength training, allowing for shorter exercise sessions.

There are guidelines for the content, intensity, frequency, and duration of physical activity in care homes, but more studies are needed to fill this gap and provide effective and efficient exercise solutions [13]. Strength training programs should be performed 2 to 3 times per week, with increment regarding exercise intensity. To optimize functional capacity, strength training should incorporate exercises that simulate daily activities, such as sit-to-stand exercises. Despite the proposed recommendations, the authors of this systematic review pointed to the need to test these recommendations in new experimental studies.

Considering the previously mentioned limitations, the need to understand the effects of physical exercise on a frail population such as institutionalized elderly, and the necessity to maintain or increase muscle mass, this study aimed to analyze the effect of a strength training program on elderly residents in care homes. It is hypothesized that a low-cost physical exercise program can increase muscle quality, specifically in the muscular endurance of the lower and upper limbs, while simultaneously improving quality of life through indirect indicators related to muscle strength.

## 2. Materials and Methods

### 2.1. Participants

This is a quasi-experimental pilot study. The a priori sample size was calculated using the G*Power 3.1 program considering the following inputs: anticipated effect size = 0.07, error probability = 0.05, statistical power = 0.95. The output indicated that 24 participants would be necessary to obtain robust statistical power to consider the results reliable. This study included a sample of 31 institutionalized older adults (20 females, 11 males) aged between 65 and 96 years (M = 81.64, SD = 8.67). Participants reported an average institutionalization duration of 2.43 years (SD = 2.20), ranging from half a year to ten years. They reported an average of 0.42 falls (SD = 0.95), totaling 9 falls, with 22 participants never having fallen, 8 having fallen at least once, and 1 participant reporting five falls.

Participants were included in the study if they met the following criteria: being institutionalized older adults (aged equal of above 65 years old); not suffering from cognitive disorders, as assessed using the Mini-Mental State Examination, with scores equal to or lower than 17 indicating dementia; having no medical contraindications for exercise; participating voluntarily; and being able to move independently. Participants were excluded from the analysis if their participation in exercise sessions was less than 75%.

### 2.2. Data Collection Procedures

Before data collection began, the present study was reviewed and approved by the Ethics Committee of the Polytechnic Institute of Leiria, with approval number 59/2022 on the 14 December 2022. The data collection procedure involved four main phases. In the first phase, an initial visit was made to the nursing homes by convenience to present the study’s objectives. Following institutional approval, residents were contacted to select potential participants, where information about the study was provided and informed consent was obtained to ensure voluntary participation and clarify any doubts. Next, an initial evaluation of the participants was conducted, consisting of sociodemographic characterization and physical fitness assessment. Finally, participants were re-evaluated one week after the intervention ended. These evaluations were carried out in the three institutions where the study took place, as well as throughout the intervention, with designated spaces provided for exercise sessions. The collected data were solely for the purpose of the study and were destroyed once its academic and research objectives were fulfilled. Participants were informed of the voluntary nature of their participation and were given the freedom to withdraw from the study at any time without facing any negative consequences.

### 2.3. Exercise Intervention

A group-based strength training protocol was implemented, with a maximum of 10 participants per group. The participants in this study were not from three different institutions. Also, the samples were heterogeneous by institution, with one institution having 16 participants. To maximize control over the training sessions and reduce the risk of potential adverse events, the training sessions were not conducted with all participants simultaneously. Instead, they were divided into groups of no more than 10 participants each.

The intervention followed the FITT-VP principles [19] and consisted of 12 weeks of training with two sessions per week, totaling 24 strength training sessions. Each training session comprised five exercises targeting different body regions. Training session 1 included the following exercises: shoulder flexion with a stick, knee extension with ankle weights, elbow flexion with a dumbbell, plantar flexion, and hip adduction with a ball. Training session 2 comprised hip abduction with a resistance band, sit-to-stand exercises, rowing with a resistance band, hip flexion with ankle weights, and knee flexion (standing, supported by a chair). The protocol involved a progressive increase in training volume by gradually increasing the training sets. In the first four weeks, each session consisted of three sets of five exercises performed for 30 s, with a one-minute rest between exercises. From weeks five to eight, each session comprised four sets of five exercises, again performed for 30 s with a one-minute rest. From week nine until the end of the intervention, each session included five sets of five exercises, performed for 30 s with a one-minute rest between exercises.

To effectively execute the training plan and ensure that the exercises were appropriately challenging, external loads were required. The necessary equipment included 1 kg and 2 kg ankle weights, closed-loop resistance bands of varying resistances, rubber resistance bands of different resistances, and foam balls. Sturdy cardboard sticks (0.60 m) from table paper rolls were adapted, and various dumbbells (1 kg, 1.5 kg, and 2 kg) were created using plastic bottles filled with sand. The availability of diverse equipment options allowed for progressive adjustments in exercise load over time. Adjustments were made when the predefined load became too easy for participants.

Training intensity was monitored using the Talk Test, which assesses the intensity of physical exertion by determining if individuals can comfortably converse while exercising [20]. This test serves as a substitute for lactate thresholds in various populations, including untrained individuals or those with chronic conditions. This simple tool was appropriate for the context of the exercise classes. During all classes, the training intensity allowed participants to maintain conversations with the physiotherapist or each other. Training sessions were held in designated spaces at each nursing home, ensuring optimal conditions for group physical exercise. Special attention was given to the area, floor safety, ease of access, appropriate temperature, and good lighting, preferably natural light. Participants were accompanied to the training location by aides and the session instructor. The training was conducted by a licensed physiotherapist with training in exercise prescription for older adults.

### 2.4. Instruments

To gather demographic data for the sample characterization, a simple questionnaire was administered by one of the researchers who interviewed potential participants after they had agreed to participate in the study and signed the informed consent form, including the following elements: age, sex, years institutionalized, marital status, and educational background. Researchers also contacted the physiotherapist or nurse responsible for the nursing home residents and consulted their medical records to obtain the history of falls and any chronic conditions or diseases the participant might have.

#### 2.4.1. Cognitive Function

The Mini-Mental State Examination (MMSE) is a test developed to assess cognitive function and distinguish individuals with cognitive disorders from those without. The MMSE consists of 30 questions that evaluate attention, memory, recall, calculation, language, and the ability to draw a complex polygon. Some advantages of the MMSE include its translation into Portuguese, validation for the elderly Portuguese population by Guerreiro [21], high acceptance levels among health professionals and researchers, its status as an objective measure of cognitive state, and its ease of administration, requiring only a few minutes to complete [22]. Cognitive decline or dementia is traditionally determined by cutoff scores below 23/24 points. However, given that most participants reported having only up to the 4th grade or less education, a cutoff score of 17 was used. Thus, individuals scoring below 17 on the MMSE were deemed ineligible to participate in the study. The questionnaire was administered at the start of the assessment along with the sociodemographic characterization questionnaire. All evaluated participants scored 17 points or higher, so no participants were excluded due to cognitive function.

#### 2.4.2. Handgrip Strength

Handgrip strength is the preferred measure for assessing overall muscle strength in clinical trials and diagnosing sarcopenia and frailty [23]. It was shown to be a good substitute for evaluating lower limb muscle strength and is much easier to perform. The test requires only a hand dynamometer, with established measurement protocols and validated cutoff values, unlike lower limb strength tests. Participants were shown how to perform the test and instructed to squeeze the dynamometer with maximum force for three to five seconds. The test was conducted with the participant seated and arm fully extended. The dominant or stronger hand was identified, and the test was performed on both sides if the participant was unsure. The test was repeated three times, with the highest value recorded. A GRIPX EH101 dynamometer (South El Monte, CA, USA) was used.

#### 2.4.3. Lower Limb Muscle Endurance

The 30 s chair stand test, part of the Senior Fitness Test battery [24], was used to assess lower limb muscle endurance. The participant starts from a seated position, arms crossed, and performs as many stand-ups and sit-downs as possible in 30 s.

#### 2.4.4. Upper Limb Muscle Endurance

The 30 s arm curl test, also part of the Senior Fitness Test battery [24], was used to evaluate upper limb muscle strength. The participant, seated, performs as many elbow flexions as possible in 30 s, starting with the elbow fully extended and holding a dumbbell (1 kg for females, 2 kg for males) in the dominant hand.

#### 2.4.5. Agility and Dynamic Balance

The Timed Up and Go test, part of the Senior Fitness Test battery [24], assesses gait speed and agility. The test involves walking a straight distance from a seated position to a cone placed 2.44 m away, rounding the cone, and returning to the seated position. Participants were instructed on the test, allowed a practice run to correct errors, and timed during the actual test. Regular footwear and walking aids were used. Faster execution times indicate better functional performance [25].

#### 2.4.6. Body Mass Index

Height was measured using a Seca 213 portable stadiometer with an integrated level (GmbH & Co. KG, Hamburg, Germany). Participants stood against a wall with the back of their head, back, and buttocks touching the wall, feet together, weight equally distributed, looking straight ahead, inhaling and holding breath. The headpiece was lowered to the highest point of the head, and the measurement was recorded. Weight was measured with a SilverCrest scale (SilverCrest, Uttenweiler, Germany) placed on a level, firm surface. Participants were weighed without shoes and coats. BMI was calculated as: BMI (kg/m^2^) = weight (kg)/height^2^ (m).

#### 2.4.7. Waist Circumference

Waist Circumference was measured using a tape measure. Participants stood with an upright trunk, relaxed abdomen, arms hanging at their sides, palms inward, head up, feet together, and weight equally distributed. Jackets were removed, and shirts were pulled up to clear the waist and abdomen. The tape was positioned around the waist, specifically between the midpoint between the lower margin of the rib cage and the iliac crest, following the guidelines outlined by the WHO [26]. Participants were instructed to exhale normally during this procedure. The measurement was taken at the end of exhalation. This process was repeated twice, with the final value being the average of the two measurements.

### 2.5. Statistical Analysis

The data were exported to the statistical program IBM SPSS Statistics v23. Descriptive statistics, including means and standard deviations, were calculated for all variables under investigation. The normality of the data was assessed using the Shapiro–Wilk test for sample sizes less than 50, while homoscedasticity was examined through the Levene test. As a criterion for accepting a univariate normal distribution, skewness and kurtosis values had to be within the ranges of ±2 and ±7 according to Cohen [27]. To explore differences between dependent variables, a paired samples t-test was used. The significance level for rejecting the null hypothesis was set at 5% for all statistical tests. The effect size, d, was calculated, with the reference values for interpretation as follows: 0.2 = small effect; 0.5 = moderate effect; 0.8 = large effect.

## 3. Results

**Figure 1:**
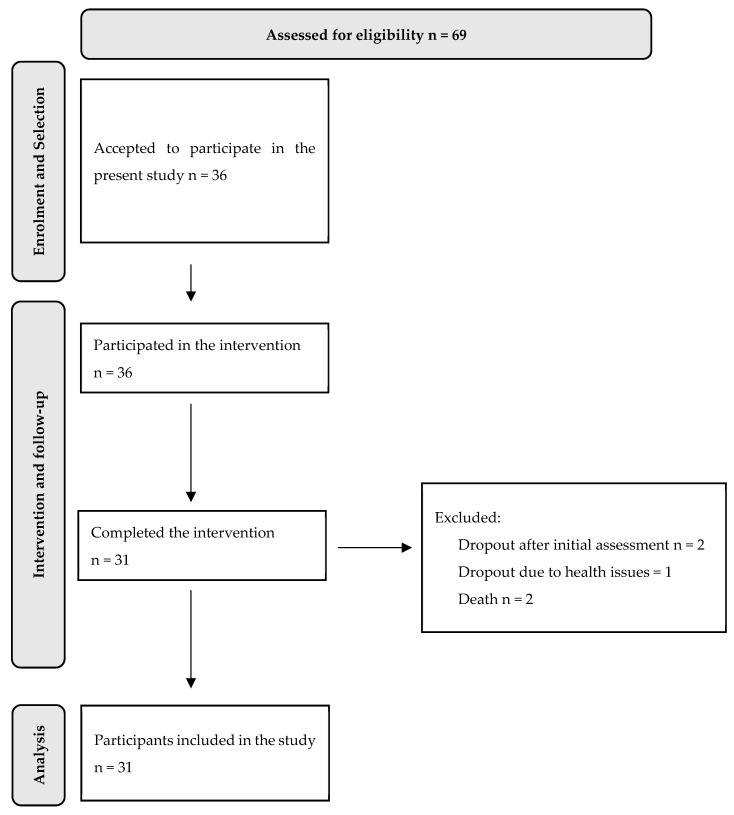
Participant flowchart.

The flowchart representing the allocation of participants in the study is presented below (Figure 1). Initially, out of 69 potential participants, 36 individuals met the inclusion criteria and were selected to participate in the study. The 33 excluded individuals either could not move independently or did not wish to participate in the exercise sessions. These falls were recorded since the individual became a resident. Subsequently, the 36 included participants were evaluated. After the evaluation, two participants left the program: one because they did not want to follow the exercise program and one due to hearing difficulties that impeded normal session functioning. During the program, two participants passed away, and one participant left due to health issues. As a result, the final count for the experimental group decreased to n = 31. Participants’ marital status included married (n = 6), divorced (n = 3), single (n = 3), or widowed (n = 19). Regarding educational background, participants reported having no formal education (n = 7), completed 1st grade (n = 4), 2nd grade (n = 1), 3rd grade (n = 9), 4th grade (n = 9), or 11th grade (n = 1). Diabetes was identified in 11 cases (34.3%), while hypertension affected 20 individuals (62.5%). Gallstones, liver congestion, atrial flutter, polycystic kidney disease, and osteoporosis were less common, occurring in one to two cases. Dyslipidemia was found in 10 participants (31.3%), and hypercholesterolemia was diagnosed in 3 cases (9.4%). Hypothyroidism and atrial fibrillation were observed in two cases (6.3%). Chronic obstructive pulmonary disease, bipolar disorder, chronic kidney disease, ischemic stroke, schizophrenia, right shoulder fracture, glaucoma, heart failure, anemia, prostate hyperplasia, dementia, esophageal hernia, cataracts, bronchitis, anxiety, obesity, depression, depressive syndrome, spondyloarthrosis, left hemiparesis, vertigo syndrome, asthma, and eczema were diagnosed in one to five cases each.

It is relevant to highlight that no emergencies or hospitalizations related to the exercise program were recorded throughout the intervention. Additionally, it is important to note that two participants passed away during the study period; however, in both cases, the cause of death was unrelated to any adverse effects of the applied exercise. In terms of monitoring the adherence to the program, we observed a 91.8% attendance rate. During the program period, no falls were reported among the participants, either during the exercise classes or outside the program. In Table 1, we can observe the results of descriptive and inferential statistics for strength and body composition indicators before and after intervention. Regarding grip strength, a small change was observed, but it was not statistically significant. In the 30 s arm curl test, there was a significant increase, reflected by the effect size. The 30 s chair stand test also showed a significant improvement in participants’ capacity. In the Timed Up and Go test, participants demonstrated a significant reduction in test completion time with a considered high effect size. As for body mass index and waist circumference, there were no statistically significant differences.

## 4. Discussion

This study aimed to examine the effect of a 12-week exercise program, specifically strength training, on physical fitness indicators in institutionalized elderly individuals. The main observations of this study were significant improvements in different physical fitness parameters, namely lower limb muscle strength, evidenced by improved performance in the 30 s chair stand test, upper limb muscle strength, shown by improved performance in the 30 s arm curl test, and agility and dynamic balance, as demonstrated by improved performance in the Timed Up and Go test. On the other hand, no statistically significant improvements were observed in handgrip strength, body mass index, and waist circumference.

In the meta-analytic review by Labott et al. [28], small statistical effects were observed in handgrip strength, which is consistent with the results obtained in the present study. However, in a study by Cadore et al. [29], a multicomponent training program composed of strength training, balance, and cognitive training was applied. The training plan began with the application of only balance and cognitive training in the first 4 weeks of the program, after which no improvements in handgrip strength were observed. However, after applying the same training plan with added strength training for 4 weeks, significant changes in handgrip strength were observed. In the study by Chen et al. [30], improvements in handgrip strength were also observed in the group that performed only strength training. Through the application of a 12-week multicomponent training program, improvements in TUG test performance were observed in the study by Izquierdo et al. [31], which is in line with the results obtained in the present study. However, in the study by Serra-Rexach et al. [32], no improvements in the agility of the participants were observed, with no improvement in TUG test performance.

In a study by Serra-Rexach et al. [32], a multicomponent training program consisting of aerobic, mobility, and strength training was applied for 8 weeks in nonagenarians. Significant improvements in lower limb muscle strength were observed in this program. In a study by Cadore et al. [29], a multicomponent training program composed of strength training, balance, and cognitive training was applied. The training plan began with the application of only balance and cognitive training in the first 4 weeks of the program, after which no improvements in handgrip strength, knee extension strength, and hip flexion strength were observed. After applying the same training plan with added muscle strengthening training for a period of 4 weeks, significant changes were observed. In another study where a 12-week multicomponent training program was also applied, improvements in the ability to stand up from a chair were observed [31]. In the study by Chen et al. [30], three types of training were implemented in elderly individuals aged 65 to 75 years with obesity: strength training 2×/week, aerobic training 2×/week, and strength training alternated with aerobic training 1×/week for each type of training. The training programs lasted for 12 weeks. In this study, significant improvements in lower limb strength indices were observed, especially in the group that performed only strength training.

In the study by Chen et al. [30], it was found that all groups experienced a slight decrease in BMI and visceral fat area, contrary to the results obtained in the present study. This difference in results may be due to the fact that the participants in the aforementioned study were obese individuals with higher BMI and a higher percentage of visceral abdominal fat, thus having a greater margin to decrease these parameters.

Comparing the results obtained with the existing literature, it is possible to verify that the increase in muscle strength happened consistently, both in the present study and in other previously conducted studies through the application of other training programs that included muscle strengthening training, proving that even in aging populations, this type of intervention promotes strength improvement [33]. Regarding handgrip strength, the literature is unclear about the effects of strength training on improving this ability, which could be explained by the fact that the test is highly dependent on the willingness to exert maximum force on the dynamometer during the test, which may not occur in older populations, influencing the result. Also, in the TUG test, there are discrepancies in the literature regarding the improvement in participants’ agility after the application of strength training. This situation may be due to differences in the content of exercises between different training plans, so it would be pertinent to compare the effects of different training plans on improving the agility of the elderly. Finally, waist circumference and BMI, which did not show improvements in the present study, contrast with other studies where improvements were indeed observed. One possible explanation for this is that BMI and waist circumference are not only dependent on the level of physical activity practiced but also, and especially, on the diet followed during the study period.

The results of the present study further suggest that performing only strength training can achieve results similar to those of a multicomponent plan, consisting of muscle strengthening exercises, balance exercises, and stretching. In a systematic review by Cadore et al. [17], various studies on the effect of different physical exercise interventions on fall risk prevention, agility, and balance are analyzed. By analyzing different types of interventions—balance training, strength training, aerobic training, and multicomponent training—it is possible to see that interventions that included strength training were the most successful. Aerobic training is presented as important for improving cardiovascular capacity; however, in the protocol analyzed, physiotherapy was performed for 1 month and strength training for 1 month before applying the exclusively aerobic protocol, raising questions about the effectiveness of each type of training performed. In turn, in this review, balance training is only effective when combined with other forms of exercise. Thus, strength training proves to be the common factor for all observed improvements, whether in programs composed solely of strength training or in multicomponent programs where this type of training is also present.

It is noteworthy that strength training in older, untrained adults presents challenges, primarily due to concerns over injury and safety. Optimal training protocols for muscle and strength development often involve higher intensities and volumes, which may pose risks to individuals with limited prior training experience and potential age-related physiological vulnerabilities such as decreased bone density or joint integrity. Therefore, initial implementations of strength training protocols in this demographic may need to be conservative, focusing initially on neuromuscular adaptations rather than maximal strength gains [34]. Research indicates that initial improvements in strength among untrained individuals are largely attributed to neuromuscular adaptations, such as enhanced motor unit recruitment and synchronization, rather than significant muscle hypertrophy [9,16]. These early gains reflect the nervous system’s ability to optimize muscle fiber recruitment patterns, thereby increasing strength without substantial changes in muscle mass. Moreover, to fully realize the potential benefits of strength training in older adults, a gradual progression to more intensive training protocols over an extended period may be recommended. Longer-term trials are essential in this context to allow for gradual progression and adaptation to more demanding training protocols. Such trials would enable participants to safely transition from initial neuromuscular adaptations to potentially more intensive strength training, provided it is carefully monitored and individualized to accommodate their specific health status and functional abilities [17]. When knowing exactly what promotes the desired adaptations in residents of institutions, it is possible to define interventions with the highest specificity possible, in this case, training programs composed entirely of strength training.

### Limitations and Agenda for Future Research

This study presents some limitations. One limitation is the absence of a control group, as only an experimental group was included. Considering the residents of the institutions where the study was conducted, all residents who met the inclusion criteria were included in the experimental group, and there were not enough residents in these institutions to create a control group. Another limitation of the present study is the lack of control over other variables that affect physical fitness, namely the levels of activity that each resident performs outside the scope of the exercise sessions conducted during the study. Furthermore, diet is a factor that can influence the variation in body mass index and waist circumference. This element should be considered in future research, given the variability from institution to institution. The population under study is one of the most fragile compared to the population of the same age group living in the community. For this reason, one possible limitation of the present study is that elderly individuals living in institutions may not be as capable of performing the physical assessment tests, which can affect the results obtained, particularly the results of the handgrip strength assessment, which are highly dependent on the maximum effort applied by the participants. The sample’s heterogeneity in terms of sex is notable. Future studies are encouraged to consider more homogeneous samples, as this characteristic may impact levels of physical fitness and neuromuscular adaptation in response to an exercise program [35].

It is suggested that more studies be conducted to evaluate the application of each type of training individually—namely aerobic training, balance training, and strength training—instead of the application of multicomponent training programs in order to understand the benefits promoted by each type of training more objectively. It is also suggested that more studies are conducted that analyze the effect of different strength training programs, composed of different exercises, evaluating their effect on this population, so that other interventions can be developed.

## 5. Conclusions

In conclusion, strength training has been shown to improve the physical fitness of institutionalized elderly individuals, specifically in enhancing the muscle strength of the lower and upper limbs and improving agility. Strength training proves to be a tool that allows for improvements in some indicators of physical fitness even among elderly individuals in more fragile conditions, such as those in institutions.

This study has several innovative aspects. It was conducted in a real-world setting rather than a laboratory, utilizing low-cost and easily accessible materials as opposed to expensive resistance machines. Additionally, this study implemented an exclusively strength-based training program, in contrast to other training models such as multicomponent or concurrent training.

## Figures and Tables

**Table 1:** Descriptive and inferential analysis.

Variables	Pre-Intervention (T1)		Post-Intervention (T2)		*t*	*p*	d
	M	SD	M	SD			
Handgrip strength (kg)	15.96	6.64	16.40	7.43	−1.023	0.314	-
30 s arm curl (repetitions)	14.42	3.58	17.68	4.33	−5.538	0.000	3.27
30 s chair stand (repetitions)	8.33	3.27	9.61	3.38	−3.587	0.001	1.98
Timed Up and Go (seconds)	22.14	13.14	18.74	11.19	4.007	0.000	4.72
Body mass index (kg/m^2^)	29.72	5.17	29.39	5.05	1.851	0.074	-
Waist circumference (cm)	104.35	10.86	103.16	11.42	1.62	0.115	-

Notes: M = mean; SD = standard deviation; *t* = *t*-test value; *p* = significance value at 0.05; d = effect size.

## Data Availability

The data utilized in this study were obtained under a specific license exclusively for the purposes of this research. The data supporting the findings of this study are not publicly available but can be requested and accessed upon reasonable inquiry.
